# The Tuberculosis Service

**Published:** 1913-09-06

**Authors:** Everitt E. Norton

**Affiliations:** Tuberculosis Medical Officer, County of Middlesex.


### THE TUBERCULOSIS SERVICE.

By EVERITT E. NORTON, M.D., D.P.H., etc., Tuberculosis Medical Officer, County of Middlesex.

Certain of the National Health Insurance sections of the National Insurance Act, 1911 (essentially characterised by economic and altruistic, as well as by humanitarian, aims), deal with the treatment of tuberculosis. They further authorise the Insurance Commissioners to retain and apply for the purposes of research certain moneys provided by Parliament.

### The Genesis of the Service.

The Act provides for insured persons, amongst other benefits, "treatment in sanatoria or other institutions, or otherwise" (in the Act called "sanatorium benefit") "when suffering from tuberculosis" or from "such other diseases as the Local Government Board with the approval of the Treasury may appoint." For the administration of sanatorium benefit Insurance Committees are required to make arrangements "with persons or local authorities," to the satisfaction of the Insurance Commissioners and subject to approval and authorisation by the Local Government Board, with a view to providing treatment for insured persons (recommended by the Committees) (i) in sanatoria or other institutions or (ii) otherwise than in such institutions. An Insurance Committee may extend sanatorium benefit to the dependants of insured persons. The terms of the Act, therefore, clearly remove any misconception that "sanatorium benefit" means solely or necessarily treatment in a sanatorium, and render it possible for the benefit to be applied in cases of "such other diseases," as may be duly "appointed," as well as in those of pulmonary or other tuberculosis.

In February 1912 a Departmental Committee (of which Mr. Waldorf Astor, M.P., was chairman) was appointed to report "upon the considerations of general policy in respect of the problem of tuberculosis in the United Kingdom, in its preventive, curative, and other aspects, which should guide the Government and local bodies in making or aiding provision for the treatment of tuberculosis . . ." Interim and Final Reports of this Committee (which has been referred to as "Astor's Committee") have been published. These concise and excellent Reports were founded upon the decision of the Committee that "any scheme which is to form the basis of an attempt to deal with the problem of tuberculosis" should, briefly, be available for the whole community, provide for preventive measures and for the detection and treatment of the disease, and make provision for research. It was added that such a scheme should unite or correlate the activities of the various bodies, authorities, and persons concerned.

The two units of the scheme recommended by the Committee for the prevention, detection, and treatment of the disease, and intended "to complete existing public health administration in respect of tuberculosis," are (i) the tuberculosis dispensary or its equivalent, and (ii) institutional treatment.

The tuberculosis dispensary (which must be distinguished from a "tuberculin dispensary") is to serve as: (i) receiving house and centre for diagnosis; (ii) clearing-house and centre for observation; (iii) centre for curative treatment; (iv) centre for examination of "contacts"; (v) centre for after-care; and (vi) information bureau and educational centre. "No single case of tuberculosis should remain uncared for in the community, and whatever services the scheme provides should be available for all cases of the disease and, as far as possible, for children as for adults": the aim should be centralisation of effort. The administration of the scheme should, the Committee considered, be undertaken by the County or County Borough Councils (exercising powers under the Insurance Act, 1911, and under previous enactments), or by Joint Committees of two or more of these bodies; in consultation and co-operation in each area with other sanitary authorities, the Insurance Committee, and voluntary organisations. With regard to Wales, Scotland, and Ireland there are certain differences, but the main principles in view are the same. It is unnecessary, even were it possible, to refer here to the Committee's very important conclusions concerning research and prevention.

### Its Relation to County Medical Officers.

The direct result of the exhaustive inquiries of the Committee was that the recommendations made ("the Local Government Boards and the Insurance Commissioners for the different parts of the United Kingdom having approved" them) have been widely adopted and that a new public service, a branch of "public assistance," has been established and now forms (in most instances) a part of the administrative department of the County Medical Officer. The medical (tuberculosis) officers are the consultants controlling the dispensary unit provided for by the scheme—the dispensary and sub-centres and their work in its entirety: their duties are, in the main, defined by the scope and aims of this dispensary unit, "the local centre of expert diagnosis and treatment," already mentioned. The responsible chief tuberculosis officer "should be independent of control by any other medical man so far as his clinical duties are concerned," but "must necessarily be in the closest touch with the general practitioners, with the responsible officials of institutions providing treatment, and with the medical officers of health." "The success of the dispensary will depend largely on how far it is the effective centre of anti-tuberculosis operations, and on the extent to which it is co-ordinated with the other elements in the scheme" and "with all agencies, voluntary or otherwise, which have an interest in tuberculous persons." The dispensary should contain a certain number of beds to permit of observation, for short periods of selected cases, to secure that careful classification of patients which is of such great importance, and to assist the medical officer in forming an opinion (in his capacity as adviser to the Insurance Committee) as to the character and duration of treatment required by patients recommended for sanatorium benefit. "The tuberculosis dispensary should constitute a centre towards which persons interested may turn for information and guidance. The clinical facts and statistics (which should be collected upon a uniform system) should be at the service of those who are engaged in research, and should be of considerable value as *data* in that connection. The information so obtained, together with the systematic training of individual patients in the measures necessary for their own treatment, should materially assist in the education of the community generally, and thus serve to promote the prevention of the disease. In addition, the dispensary should become the centre of medical education," and of "the special training of medical practitioners and nurses."

New Openings for Medical Men.

Before making further reference to the medical (tuberculosis) officer in charge of the dispensary unit it may be well to point out that the institutional and domiciliary treatment necessary under the scheme will engage the services of an increasing number of medical men. Sanatoria, hospitals, institutions for children and for the treatment of non-pulmonary tuberculosis will rapidly increase in number and in importance. In the opinion of the Departmental Committee a sanatorium of 100 beds requires one medical officer, and one of 200 beds two medical officers, in addition to the medical superintendent; "the salaries should be such as to secure men possessing the requisite ability; in order to attract the right type of men as medical superintendents it will usually be found necessary to offer a salary of not less than £500 a year, with house and with prospects of increase." Those who intend to fit themselves to become candidates for such appointments must be prepared to prove the possession, upon the foundation of a sound general medical education, of special training in tuberculosis and its treatment, and in hospital administration; suitable experience being obtainable only in resident appointments in properly conducted hospitals or other institutions.

Frequent reference was made by the Committee to, and great stress was laid upon, the importance of the share of the general practitioner in the working of the scheme. The first knowledge of illness in almost every case amongst the various classes of patients will be through him, and upon him will usually fall the duties of "notifying" cases of tuberculosis and giving a first certificate for production to the Insurance Committee: as a result, in great measure, of his intervention, an increasing proportion of incipient, latent, and suspected cases will attend for examination, or be kept under observation, at the dispensary. It is desired to "enlist the hearty co-operation and stimulate the interest of the general practitioners," who have exceptional opportunities for "securing the early recognition and methodical treatment of tuberculosis," as well as promoting "the effective after-care of cases and encouraging . . . healthy habits of life." The Order of the Local Government Board, dated July 26, 1912, approves certain regulations for domiciliary treatment and defines the position and duties, relating thereto, of the general practitioner. The present position as to payment is well known, and it is unnecessary to refer to it.

A New Class of Appointments.

The appointments which constitute a class newly established under the scheme are those of medical (tuberculosis) officers and their assistants. The work, although resembling in some details that carried out by the medical men attached to the previously existing tuberculosis dispensaries, is new work, and is still in course of organisation and development. To quote again from the Reports of the Departmental Committee: "The one essential of the chief medical officer" (himself the "essential element which must always be present") of the dispensary is that he shall be "a first-class clinician, with special training in tuberculosis and with capacity for organisation, . . . a whole-time officer whose duties will not bring him into competition with the other medical men in the district," and one whose age and attainments will enable him "to command the confidence and co-operation of the general practitioners." He should, "wherever practicable, act in a consultative capacity as the expert adviser to the Insurance Committee concerning the character of the treatment of cases recommended for sanatorium benefit, his aim being to avoid unnecessary interference" in his relations with the practitioners, who will in many cases carry out the necessary home treatment in consultation with him. He should "himself treat cases for which special skill and experience are required." It is one of the first principles of the scheme that he shall have absolute freedom in deciding upon the form of treatment advised and in many cases upon the details of treatment—the administration of tuberculin, of course, included. The Committee's Reports anticipate the need for also providing "expert advice in surgical, dental, and other cases where difficulties may arise." In order to obtain the services of "the right type of men, suitably qualified and experienced and of the requisite abilities, as medical (tuberculosis) officers, and with a view to effectiveness and economy, it will usually be found necessary to offer a salary of not less than £500 with prospects of increase." "Whilst not desiring to lay down any hard-and-fast conditions, the Committee are of opinion that preference should be given to registered medical practitioners . . . not less than twenty-five years of age, who have held house appointments for at least six months in a general hospital in addition to a similar period of attendance at a special institution for the treatment of tuberculosis. They should be also competent to supervise such laboratory work as may be necessary." The Committee recommend that all "appointed after January 1915 should be required to give satisfactory evidence of having, subsequently to qualification, devoted at least six months to special training in tuberculosis, and at least eighteen months to general clinical work, of which six months should have been spent as resident in a hospital for general diseases or other hospital not confined to the treatment of tuberculosis." Salaries of chief officers are usually £500* and of assistants about £350 per annum. The officers are required to devote their whole time to the duties. Travelling expenses are paid, a motor-car being in some cases provided or maintained. Clerical assistance is arranged for.

The appointments, although made with the sanction of the Local Government Board and the Insurance Commissioners, are terminable upon three months' notice on either side.

The expenditure of public money now being devoted to tuberculosis must have an influence upon education and post-graduate study. The Departmental Committee, recognising this fact, desire students and practitioners to acquire familiarity with methods of diagnosis and treatment by attendance at dispensaries and institutions and by associating these with medical schools.

* Discussions have arisen as to instances in which a lower salary has been offered. Whilst it is open to authorities to offer, and to medical men to apply for or accept, appointments upon such terms, this is the *minimum* salary recommended in the Reports of the Departmental Committee and deemed adequate by the B.M.A. in the case of a chief and responsible officer.

